# Risk justice: Boosting the contribution of risk management to sustainable development

**DOI:** 10.1111/risa.14157

**Published:** 2023-05-21

**Authors:** Mathilde de Goër de Herve, Thomas Schinko, John Handmer

**Affiliations:** ^1^ Risk and Environmental Studies Karlstad University Karlstad Sweden; ^2^ Centre for Research on Sustainable Societal Transformation (CRS) Karlstad University Karlstad Sweden; ^3^ Centre for Societal Risk Research (CSR) Karlstad University Karlstad Sweden; ^4^ International Institute for Applied Systems Analysis (IIASA), Population and Just Societies Program Laxenburg Austria

**Keywords:** disaster risk management, flood risk governance, risk justice, sustainable development

## Abstract

Comprehensively addressing different aspects of justice is essential to enable risk management to contribute to sustainable development. This article offers a new conceptual framework called risk justice that comprises procedural, distributive, and corrective justice in four dimensions related to sustainable development: social, ecological, spatial, and temporal issues. Risk justice is defined as the quality of being fair and reasonable while governing and managing a possible negative event. After explaining the conceptual framework, a detailed content analysis of two international guidelines for disaster risk management (the Sendai Framework for Disaster Risk Reduction 2015–2030 and the European Floods Directive) illustrates the analytical potential of the risk justice framework. Findings show strong emphasis on social and spatial aspects of distributive and procedural justice in the two documents, whereas considerations of corrective justice and temporal and ecological issues are scarce or indirect. This may result in conflicting impacts of disaster risk management on sustainable development. Therefore, discussing risk management with a risk justice viewpoint while elaborating guidelines or choosing risk management strategies provides new avenues for sustainable development and facilitates transparent trade‐offs. Our risk justice framework enables risk practitioners and researchers to reflect systemically about justice in risk management in different risk contexts and can be used both as a proactive and as a retrospective tool.

## 1 INTRODUCTION

Two of the global challenges the world is facing are sustainable development and increasing inequalities, and these challenges are intertwined in the sense that justice is a key aspect of sustainable development. In addition, as risks are affecting the durability of the activity they threaten, it is not surprising that risk management is an essential part of sustainable development. Yet, conflicts arise when managing risks because of perceived or real injustices, and therefore, one needs to think concretely about justice in risk management to contribute effectively to sustainable development. In other words, on the one hand, risk management is important to build a path toward sustainable societies (Hunjra et al., 2022; Izumi et al., 2020; Šakić Trogrlić et al., 2022), and on the other hand, justice is critical in risk management (Adger & Nelson, 2010) and key for successful sustainable development (Agyeman, 2013). Yet, no umbrella framework in the literature addresses the different justice issues related to the various facets of sustainable development within risk management (de Goër de Herve, 2022). That is why this article presents a framework called *risk justice*. The framework itself is meant to be generally applicable to all types of risks, as defined later, and the second part of the article illustrates its application in the context of disaster risk management specifically.

The risk justice framework can be employed for several purposes by many stakeholders, such as risk managers, decision‐makers, evaluators, and researchers. It may be used in practice either as a forward‐looking (proactive) tool when deciding about new risk management strategies, or as a backward‐looking (retrospective) tool when judging past and current strategies. The article provides examples of the retrospective application.

To put it another way, our goal is to elaborate a generally applicable and multidimensional risk justice framework that aims at facilitating considerations of fairness issues within risk management in order for it to contribute to sustainable development. The application provided as an example shows its analytical potential with the document analysis of some international disaster risk management guidelines.

Such conceptual work requires not only examples but also clarification of the keywords (Jaccard & Jacoby, 2020), which are developed in the following.

In order to define risk justice, we decided to go back to linguistic definitions to explore the concepts of risk and justice, drawing on standard dictionaries. A risk is “the possibility of something bad happening” (Cambridge Dictionary, n.d.‐b) or “the possibility that something unpleasant or dangerous might happen” (MacMillian Dictionary, n.d.‐b). We note two main characteristics of risk from these definitions: the uncertainty of the event happening and the potential for negative consequences. More elaborated definitions are presented in the scientific literature (see for instance Haimes, 2009) and given by organizations that work with the concept (see e.g., ISO (2018) for a standardized understanding of risk in organizations; and SRA (2018) for an overview of several qualitative definitions and risk metrics). Turning to the concept of justice, it is defined as “fairness in the way people are dealt with” (Cambridge Dictionary, n.d.‐a) or “the fair treatment of people” (Oxford Learner's Dictionaries, n.d.). Other definitions also include the idea of reasonableness: Justice is “the fact that something is reasonable and fair” (MacMillian Dictionary, n.d.‐a) and “the quality of being fair or reasonable” (Oxford Learner's Dictionaries, n.d.). In summary, *we define the concept of risk justice as the quality of being fair and reasonable while governing and managing a possible negative event*.

We therefore need to provide specific definitions of risk governance and management. Risk governance is “the application of governance principles to the identification, assessment, management and communication of risk. Governance refers to the actions, processes, traditions and institutions by which authority is exercised and decisions are taken and implemented” (SRA, 2018, p. 8) and risk management is the “activities to handle risk such as prevention, mitigation, adaptation or sharing” (SRA, 2018, p. 8).

It is important to clarify here that a risk might offer benefits that explain the willingness to take it, but there are always negative consequences if the risk materializes (SRA, 2018). We note as well that the definitions of justice refer to fairness, which itself is not defined. This is so because what is considered fair depends on many factors, including cultural and historical contexts, types of risks, and types of management strategies (see for instance de Goër de Herve, 2022, concerning the various meanings of fairness in flood risk management). Even if there is no agreement on what is just or not, Johannesson et al. (2022) suggest that it is possible to agree on a justice assessment framework. The risk justice framework offers such a structure for justice assessment in the context of promoting the contribution of risk management to sustainable development.

Interestingly, there is no clear definition of sustainable development that is broad enough to integrate the many aspects of the concept in online dictionaries. The only agreement in the scientific community is that there is no consensus on the definition, but most scientific discussions include at least one of the following three elements: targets, territories, and time (Martinuzzi & Meyer, 2016). *Targets* group the different issues related to sustainable development, such social and ecological ones. The Sustainable Development Goals (General Assembly Resolution, 2015, 70/1) are an example of this understanding of sustainable development. *Territories* encompass different spatial levels and highlight that actions taken in one place should not hinder sustainable development in other places. *Time* is about the long term and the belief that current actions should not limit the well‐being of future generations. This is a key part of the definition of sustainable development in the Brundtland Report (World Commission on Environment and Development, 1987) that concerns the capacity of future generations to meet their own needs. Fairness issues emerge in relation to these three elements of sustainable development. Even if some have argued that sustainable development focuses on economic growth, whereas the concept of sustainability encompasses a complex system analysis of nature and human relationships (Ruggerio, 2021), we notice that the Sustainable Development Goals, a broadly accepted conception of sustainable development today, are not focused on economics only (General Assembly resolution, 2015, 70/1). As the notion of “sustainability” gives the impression of a fixed target and leaves out the idea of a perpetual evolvement toward better well‐being, which the word “development” includes, we have chosen to use the notion of “sustainable development” in this article.

For risk management to contribute to the different “targets” of sustainable development, the concept of justice has to be extended from a purely anthropocentric perspective (fairness between people, which includes social and economic issues) to a larger understanding that includes natural systems (fairness between both humans and nonhumans, which includes environmental and ecological issues). As these entities can be in different places, the fairness between them relates to the “territories” aspect of sustainable development. In addition, the entities can also be living at different moments in time, and thus, the fairness between them affects the “time” element of sustainable development. The many meanings of sustainable development increase the likelihood of conflicting goals, and therefore, the idea of reasonable treatment of entities included in the concept of justice is also important for being able to deal with necessary trade‐offs.

As mentioned previously, the second half of this article presents an application of the framework in the specific case of disaster risk management. Disaster risks were selected among the many possible examples of risks because their reduction is essential for sustainable development (United Nations, 2015). A disaster risk is a combination of the potential negative consequences of a hazardous event given the degree of exposure and vulnerability of the people and assets present in a place at a particular moment. Numerous disasters (such as heatwaves, fires, and floods) in the last years have occurred in Europe and around the world (Bevere & Remondi, 2022), making an increasing number of people experience their direct effects. Disasters uncover inequitable situations leading Thomalla et al. (2018) to call for moving from “current development patterns that increase, create or unfairly distribute risks, to forms of development that are equitable, resilient and sustainable” (p. 1). More than 10 years ago, the Intergovernmental Panel on Climate Change introduced the idea that equity is an essential part of disaster risk management in the face of climate change (IPCC, 2012) and therefore necessary for sustainable development. Consequently, disaster risk management is a suitable illustration for applying the risk justice framework.

The article is divided into five sections. Section 2 describes the methods for theory‐building and document analysis. The framework is presented and described in Section 3, and Section 4 details the illustration of its application as a retrospective analytical tool. To conclude, Section 5 discusses the findings and the risk justice conceptual framework.

## 2 METHODS

### 2.1 Theorization

The present article follows a theory‐building approach that gathers information from previous scientific publications in order to synthesize multiple theories into a unified framework, which is one of the main ways of making a theoretical contribution according to Jaccard and Jacoby (2020). “Constructing a theory is more like crafting an elegant ensemble of logically connected ideas that depict the world and allow knowledge to leap forwards” (Series Editor's Note by T.D. Little, Jaccard & Jacoby, 2020, p. vii). To do so, we connect and bring together ideas from relevant existing literature. The resulting risk justice framework is at the crossroads of two main categories of conceptual works, according to the typology developed by MacInnis (2011): revising and delineating. Among these categories, there are a number of types of tasks. The article uses two of these: First, envisioning tasks that characterize different justice aspects that are sometimes not labeled as such and second, explicating tasks that show how the dimensions are relevant when taken together as a whole. MacInnis (2011) states that “conceptualization is a process of abstract thinking involving the mental representation of an idea” (p. 140) and that it is “critical to vitality of academic fields” (p. 150).

The premises of the framework have been presented during research seminars and conferences (e.g., de Goër de Herve, 2021), and an informal interactive review of the work in the form of discussions with subject‐matter specialists was conducted to challenge its content and its relevance. An initial version focusing on flood management, built following a literature review on justice considerations within flood risk management, has been published under the name of “flood risk justice” (see de Goër de Herve, 2022). The review led to improvements of the framework while generalizing it to various types of risks, with for instance the addition of a third meta‐dimension, corrective justice.

### 2.2 Document analysis

We test the framework through the analysis of two documents. The Sendai Framework for Disaster Risk Reduction 2015–2030 (United Nations, 2015), hereafter “SFDRR”, has been selected for analysis as an international guideline for disaster risk management. It was released in 2015, the same year that two other key international agreements for sustainable development were agreed upon: the Sustainable Development Goals and the Paris Agreement. In addition, the European Floods Directive (Directive 2007/60/EC), hereafter “FD”, has been selected as it is an example of an international binding agreement between the European Union Member States on flood risks, which are one of the risks concerned by the SFDRR. Floods are among the most frequently occurring disasters with globally the greatest impacts and largest numbers of affected people (CRED, 2022; Delforge et al., 2022). Appendix A summarizes the two documents.

The document analysis was processed in two parts: a count of the word justice and some synonyms and their antonyms (we searched for “just,” “fair,” “equit,” and “equal” in the text) and a content analysis with coding based on the different dimensions of the framework. The rather limited appearance of justice‐related words during the word count (see results in Table 2) called for an in‐depth qualitative content analysis, as some elements can be related to justice issues without being labeled as such in the texts. The content analysis was proceeded in two steps: First, general codes related to the several dimensions of risk justice were applied to the qualitative data. Second, the codes were fine‐tuned based on the first step analysis, allowing for a more precise second content analysis to elaborate the preliminary findings. The final list of codes is presented in Appendix B. This was undertaken by the lead author with results cross‐checked by the coauthors to strengthen their validity.

## 3 RISK JUSTICE

### 3.1 Conceptual framework

**FIGURE 1:**
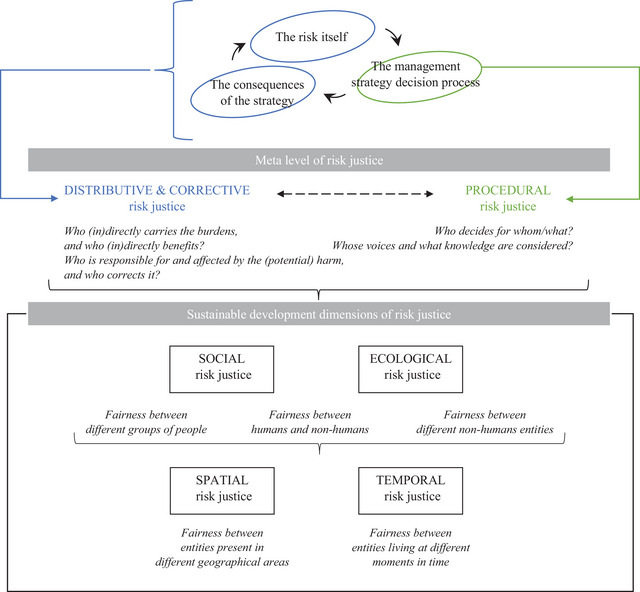
The conceptual risk justice framework.

The conceptual risk justice framework considers distributive, corrective, and procedural justice in four dimensions (social, ecological, spatial, and temporal) related to sustainable development. Risk governance and management can be described in a very simplified way as a risk that is identified, a choice of a management strategy, and the consequences of this strategy once implemented, which should have an impact on the risk itself (among other consequences) as shown at the top of Figure 1.

Distributive justice, which is the fairness between those who directly and indirectly benefit and those who directly and indirectly carry the burdens (de Goër de Herve, 2022), applies both to the risk itself (e.g., Busby & Sedmak, 2011; Dietrich, 2021) and to the management strategy (e.g., Kaufmann et al., 2021; Thaler, 2021). In essence, it can be addressed by asking the following questions: Who is impacted positively and negatively by the risk? Is that fair? Who is impacted positively and negatively by the management strategy? Is that fair? In many cases, distributive justice can be interlinked with corrective justice, which is about establishing responsibilities for the harmful event. Corrective justice raises the questions of who is responsible for the harm? Who is affected by it? Is that fair? Who should correct the harm, and whether that is fair?

The theoretical distinctions between corrective and distributive justice are discussed in philosophy (see for instance Perry, 2010), and according to Weinrib (2002), a classical fundamental difference comes from Aristotle's distinction: corrective justice “focuses on whether one party has committed and the other has suffered a transactional injustice” (p. 349), whereas distributive justice “deals with the distribution of whatever is divisible […] among the participants in a political community” (p. 349), and therefore, “there is a conceptual difference between the correlative logic of corrective justice and the comparative logic of distributive justice” (p. 355). In Aristotle's understanding, corrective justice can happen between two parties only because one is responsible for the loss of the other one, whereas distributive justice can happen between an unlimited number of parties (Weinrib, 2002). Yet, in the risk justice framework, we extend this limited understanding of corrective justice by considering that corrective actions may be taken by a third party, for instance when a public policy aims at remediating polluted land in order to restore its ecological value, whereas those responsible for the pollution cannot be identified or are otherwise unable to rectify the wrongdoing. Lazar (2008) contends that the harm can be corrected by a different agent than the harmer, as long as it is indeed rectified.

In practice, it might be easier to discuss distributive issues concerning the current and future distributions of resources, and corrective ones in the case of past and historical situations that lead, or can lead, to loss and damages. For instance, Wallimann‐Helmer et al. (2019) compare compensatory and distributive justice for climate‐related loss and damage and conclude that in practice, compensatory justice (which is one possible application of corrective justice) applies for identified harm that has already happened, whereas distributive justice can be used to foster fairness when implementing new strategies.

Procedural justice, which is about who takes the decision and whose voices and what knowledge are considered during the decision process (de Goër de Herve, 2022), is key when choosing a management strategy (Figure 1). It asks the following questions: Who makes the decision? Is that fair, especially when compared to who is affected? Whose voices are heard, and whose knowledge is considered? What information is taken into consideration? Is that fair? Therefore, procedural justice is both about the participation of the relevant stakeholders and the information used to make the decision.

Distributive and corrective justices are linked to procedural justice, and the other way around. Some argue that there cannot be serious distributive justice without procedural justice, and that there is a need for the fair distribution of resources, such as time, money, and knowledge, in order to foster procedural justice (Begg, 2018). This meta‐level of risk justice is visible in the middle of Figure 1. Yet, the causal links among the different forms of justice vary widely (O'Hare & White, 2018). In addition, there is often a need for justice of recognition in order to support procedural, distributive, and corrective justice. Indeed, the recognition of those affected by the risk or the harm, and those affected by the management strategies, leads to better delimitations of who and what should be included in the decision‐making process (Kaufmann et al., 2021).

The innovative aspect of risk justice is that distributive, corrective, and procedural justices are considered in terms of four dimensions related to sustainable development: social, ecological, spatial, and temporal justice, as shown at the bottom of Figure 1. Social justice is the fairness among different groups of people, and ecological justice is the fairness between humans and nonhumans, as well as among different nonhuman entities. They should both be analyzed together with spatial–temporal considerations. Spatial justice is the fairness between entities present in different geographical areas, and temporal justice is the fairness between entities present at different moments in time. As human and natural systems are interconnected and various spatial–temporal scales are included, the different dimensions overlap. They are relevant for the different facets of sustainable development, in particular the target integration of social and ecological justice, the territorial integration for spatial justice, and the time integration for temporal justice. Therefore, risk justice is about both humans and nonhumans, here and now as well as elsewhere and in the future.

The four sustainable development dimensions included in the risk justice framework build on existing theories in the justice literature (see Table 1). The social dimension reflects the broad frames of social and intragenerational justice, as well as environmental justice, which focuses on social issues in the case of environmental risks and resource management (Schlosberg, 2007). The ecological justice dimension of risk justice is inspired by what is also called ecological justice, which is the fairness between human and nonhuman systems and entities, as well as multispecies justice, which is the fairness among different species. In the context of risk justice, the spatial dimension includes issues raised by climate justice for climate‐related risks, such as the responsibility for and impacts of climate change, as well as the capacity to cope with impacts between different countries or regions of the world. Environmental justice also offers spatial insights about environmental risks, and international justice starts out from national borders to discuss fairness. The understanding of spatial justice in the context of risk justice also includes attention to other spatial levels such as different places located within the same country. Temporal justice extends the idea of intergenerational justice to include nonhuman entities as well. It can also reflect fairness issues between human and nonhuman entities who have lived in the past, and the ones living today. We understand temporality broadly, covering different points in time in the short, medium, and long‐term.

**TABLE 1:** Justice frameworks inspiration

Dimensions of risk justice	Inspired by existing justice frameworks in the literature
Social	‐Social justice ‐Intragenerational justice ‐Environmental justice
Ecological	‐Ecological justice ‐Multispecies justice
Spatial	‐Spatial justice ‐Climate justice ‐Environmental justice ‐International justice
Temporal	‐Intergenerational justice

### 3.2 Details for distributive justice in the four dimensions

**FIGURE 2:**
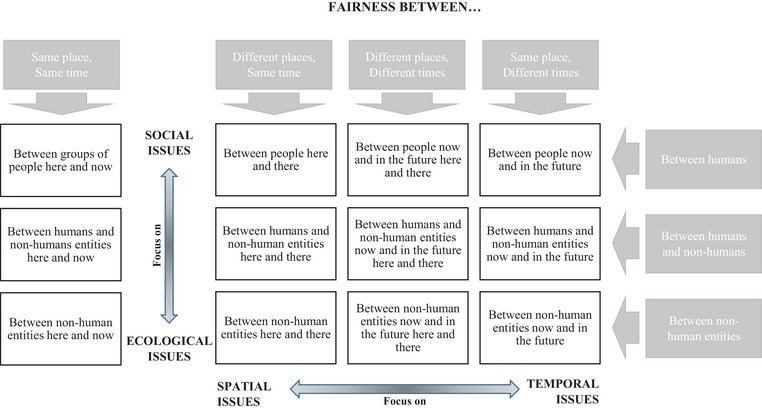
Detailed components of distributive risk justice.

Figure 2 presents a matrix that encompasses the scope of distributive risk justice. The rows detail fairness issues between the stakeholders: different human groups and different nonhuman entities. The columns add the spatial–temporal frames to discuss the distribution of the impacts of the risk and the management strategy among these actors.

Each management strategy may be discussed within each of the boxes appearing in Figure 2. Is the specific risk and/or the strategy to handle it concerned with distributional issues of any box and all the other boxes? If yes, is the situation just, given what is considered fair in the context?

### 3.3 Details for corrective justice in the four dimensions

**FIGURE 3:**
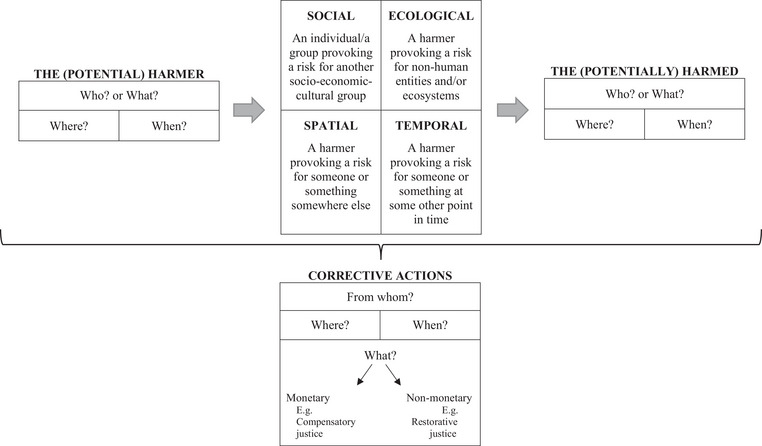
Corrective risk justice mapped.

Corrective justice establishes responsibilities for harms and their corrections between the different groups and entities. “Harm is a damage to a person's interests” (Lazar, 2008, p. 356) or, in the case of risk justice, a damage to the interest of an entity, whether it is human or nonhuman. Yet, Lazar (2008) states that money, even if it may be “a means for furthering our interests” (p. 356), has certain limitations for correcting the harm, notably because some harm can never be compensated, especially if it cannot be undone, and because some situations cannot be valued in money: They are incommensurable. Therefore, corrective justice in risk management cannot be reduced to monetary compensation only but can include other actions such as restoration processes. So corrective actions group monetary and nonmonetary strategies as shown at the bottom of Figure 3. Compensatory justice refers to “the provision of resources to a victim with the goal of minimizing or reversing the impact of harm done by the injustice” (Mullen & Okimoto, 2015, p. 478). As it is about a transfer of resources, for instance money, this type of justice usually applies to the social dimension of risk justice, the fairness between humans, and can apply in various spatial–temporal scales. Restorative justice focuses broadly on the recovery and healing process after harm has been done; it “calls for a repair of harms done to communities and the environment” (Spurlock et al., 2022, p. 2). It can apply to humans as well as nonhumans (e.g., the restoration of ecosystems after an environmental pollution). Restoration processes may take time and therefore include a temporal dimension.

**FIGURE 4:**
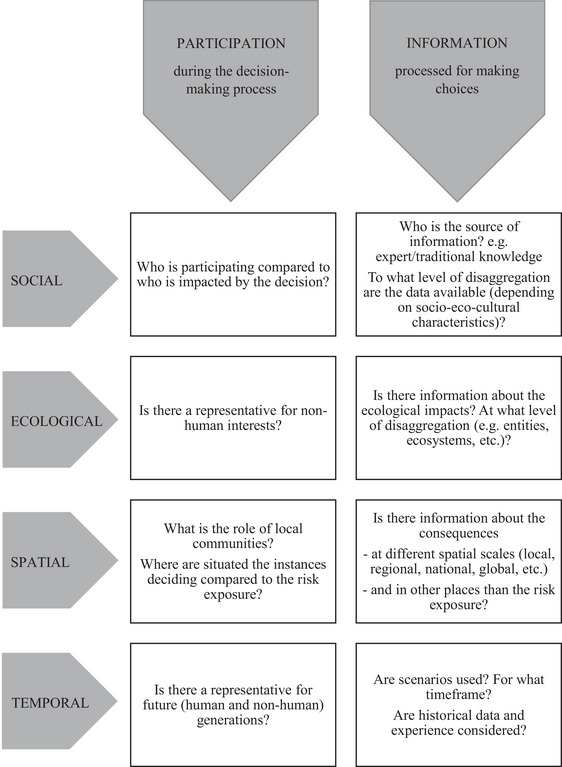
Non‐exhaustive list of procedural justice concerns in the four sustainable development dimensions.

### 3.4 Details for procedural justice in the four dimensions

As summarized in Figure 4, procedural justice concerns both the question of who participates during the decision‐making process and the question of what information is considered when choosing what strategy to implement. Procedural justice in the social dimension is most often discussed in terms of participation in and access to the decision‐making process (e.g., Adger & Nelson, 2010). Who can participate in the decision‐making process? Who is given the opportunity, and who does it in practice? This discussion also raises the trickier question of who should be invited to participate: experts in the area of study who have theoretical knowledge, or people exposed to the risk who have knowledge derived from experience but may be biased by inaccurate perceptions? Moreover, a critical issue is to consider whether or not increasing the number of participants in the decision‐making process always increases fairness. Previous research has shown that taking part can sometimes turn into a burden for the participants and result in a transfer of responsibilities rather than power (Begg, 2018).

In addition, the other dimensions of sustainable development bring challenging decisions: how to enable the participation of future generations or nonhumans? Even more challenging is how to enable the participation of nonhumans that will live in the future? Hypothetical reasoning can guide decisions about risks affecting people who cannot take part in the decision procedure such as future generations (Hermansson, 2010). As a minimum, their interests should be considered during the decision‐making process, for instance through the invitation of representatives of specific NGOs (see Pahl‐Wostl et al. (2013) referred to by Begg (2018)). Among the various actors that should be included in justice assessments, Johannesson et al. (2022) suggest a “justice caller,” who would present “a justice claim on behalf of an actor who cannot exercise her rights directly” (p. 7). They state that “with relevance to sustainable development and climate justice, we note that some actors may not be able to come in contact with the system directly. […] Separate individuals and activist groups may act as justice callers for future generations who do not yet have a voice in climate issues or on the behalf of ecosystems that cannot speak for themselves by definition” (p. 7).

When it comes to what knowledge is considered, and therefore, what information is processed during the decision‐making process, including a social dimension can call for disaggregated data in order to analyze the potential impacts on different communities. Including an ecological dimension requires information about the consequences of the potential strategies on nonhumans, and including a temporal dimension can be represented by the consideration of long‐term scenarios and the inclusion of foresight methodologies to support decision‐making. The spatial dimension requires a check on the potential consequences of the actions on different geographical areas and not only the one where the hazard and/or the strategy is located.

As mentioned in the introduction, the risk justice framework can be applied to very different types of uncertain and negative events. The next section uses it to look specifically at disaster risk management.

## 4 ILLUSTRATION: ANALYSIS OF DISASTER RISK MANAGEMENT INTERNATIONAL GUIDELINES THROUGH THE RISK JUSTICE FRAMEWORK

To illustrate the analytical potential of the risk justice framework, we applied it to official documents used as compulsory or voluntary guidelines for disaster risk management. The SFDRR (United Nations, 2015) is an international voluntary agreement on how to support disaster risk reduction worldwide. It clearly states that disaster risk management is a way to contribute to sustainable development. We present the findings of the analysis of the SFDRR through the risk justice lens in the first part of the results section. Then follows a more specific example focusing on flood management. The basis for the establishment of flood management plans and their implementation within the European Union is the FD (Directive 2007/60/EC), which is analyzed in the second part of the result section.

Table 2 shows a very limited mention of justice or synonyms and antonyms in the two documents, as well as in the Water Framework Directive (Directive 2000/60/EC) of the European Union, hereafter “WFD”, a key directive to which the FD often refers. The results presented thereafter are therefore based on the in‐depth‐content analysis described in Section 2. We present only a summary in the article because of length limitation, and the longer version can be found in the material.

**TABLE 2:** Word count of justice and synonyms/antonyms

	Sendai Framework for Disaster Risk Reduction 2015–2030 (SFDRR)	EU Floods Directive (FD)	EU Water Framework Directive (WFD)
*just*	0	0	0^a^
*fair*	0	1 (“fair” sharing of responsibilities)	0
*equit*	3 (“gender equitable”: once in the core text, once in the index, and once in the chart)	0	1 (“equitable” water use)
*equal*	2 (“inequality” as a driver of disaster risks: once in the core text, and once in the index)	0	1^b^ (the parties are “equal” in the decision‐making process…)

*Note*: Only the words related to justice appear in the table (excluding a) “adjusted”; “justified”; “just” as a meaning of “only”; b) “equally” in the meaning of “as well”; “equal” for a color code).

### 4.1 Sendai Framework for Disaster Risk Reduction 2015–2030 (SFDRR)

The SFDRR includes several elements of procedural and distributive justice but limited consideration of corrective justice. A more detailed description of the issues presented in the SFDRR through the risk justice lens is available in the Supporting Information section. In summary, the justice‐related elements of the SFDRR contain a strong focus on social and spatial sustainability with many explicit statements in these contexts, whereas less emphasis is put on the temporal dimension, and the ecological dimension is relatively underrepresented.

#### 4.1.1 Social issues

The SFDRR argues that the management of disaster risks should be preventive, people‐centered, and assist those who are disproportionately affected by disasters (such as women, the elderly, migrants, and indigenous groups). The main strategy is to invest in the resilience of people and communities according to their respective vulnerabilities and needs in order to reduce potential losses and damages, with a focus on tackling root causes of disaster risks by for instance investing in reducing poverty and hunger, and improving educational, health, and telecommunication infrastructure. Clear tasks and responsibilities for risk management should be assigned to relevant stakeholders, which is connected to the allocation of needed resources. The SFDRR highlights procedural elements and encourages whole‐of‐society engagement in the decision‐making process, with special attention to giving voice to people disproportionally affected by disasters. The process should be empowering and inclusive with accessible and nondiscriminatory participation. Decision‐making collaboration includes all stakeholders in society: public and private, at different institutional levels and in different sectors. Scientific, traditional, and indigenous knowledge, as well as knowledge from experience, must inform the decision‐making process. Disaggregated data (i.e., by sex and age) helps with identifying the needs of different affected people. Corrective justice aspects refer to the need for accountability for disaster risk creation at all levels. The SFDRR also suggests the promotion of mechanisms that can be interpreted as international corrective measures such as risk transfer and insurance.

#### 4.1.2 Ecological issues

According to the SFDRR, the management of disaster risks aims, among other goals, at protecting environmental assets and ecosystems, with a focus on investing in environmental resilience, which necessitates for example resource management and biodiversity promotion. The choices should be made considering the vulnerability and exposure of the environment and the effects of disasters on ecosystems and environmental heritage. However, suggestions of corrective measures for ecological purposes are missing.

#### 4.1.3 Spatial issues

The guidelines for each priority in the SFDRR are presented for different spatial levels, and there is a section dedicated to “International cooperation and global partnership.” Cooperation among various spatial levels and international collaboration mechanisms are essential for coherent management. In general, local characteristics of disaster risks should be taken into consideration during the decision‐making process. There is an emphasis on the “developed” countries helping “developing” countries, and this help should be based on the needs and priorities identified by the beneficiaries themselves. The SFDRR acknowledges that some countries face specific challenges because of higher vulnerability and hazard levels and are therefore disproportionally affected by disasters. Finally, the SFDRR encourages some actions that can be interpreted as corrective, for example strengthening the resilience of affected people and that of host communities, and relocating public facilities and infrastructure to places outside the hazardous areas in the post‐disaster reconstruction process.

#### 4.1.4 Temporal issues

According to the SFDRR, the management of disaster risks should first and foremost prevent future losses by focusing on prevention and preparedness. This includes an emphasis on investment in resilience through tackling root causes of disaster risks and other strategies such as building back better. The participation and leadership of children and youth are strongly encouraged as they are agents of change. Information about the factors and scenarios for disaster risks in the medium and long term should be considered, in particular in the light of climate change. To make decisions, it is also important to learn from past programs and disaster reviews. There is no specific mention of corrective actions related to temporal justice.

The SFDRR is an international voluntary agreement that advises disaster risk reduction in a broad sense. The next part focuses on one of these disaster risks, namely floods, within the European Union spatial context.

### 4.2 European Floods Directive (FD)

The FD touches upon all elements included in the risk justice framework, although some are addressed only indirectly through reference to other documents; such as the WFD and the Charter of Fundamental Rights of the European Union (2012/C 326/02)^1^. The FD mentions the contribution of flood management to sustainable practices, through environmental protection, sustainable land use practices, and sustainable human activities. A more detailed analysis of the FD elements is available in the Supporting Information section.

#### 4.2.1 Social issues

The FD recommends an active involvement of all interested parties in the production and update of management plans through public information and consultation. All costs and benefits of the strategies must be considered in decision‐making. The plans should be inspired by best practice cases and best available technology and adapted in line with scientific and technical progress. The measures should prevent damage, and, if possible, reduce the likelihood of flooding. The FD argues that there must be a “fair sharing of responsibilities” (p. 28) for measures jointly decided for the common benefit of the European Community, in light of the solidarity principle. As the FD respects the Charter of Fundamental Rights of the European Union, it must consider the right to life for everyone (article 2), the right to property (article 17), and the prohibition of discrimination (article 21). The FD also recognizes that some human activities and climate change contribute to the harm generated by floods. In the case of a disaster event, the European Solidarity Fund can grant rapid financial assistance to help people return to preflood conditions, which is a corrective measure. In general, the WFD recommends basing corrective measures on the polluter‐pays principle.

#### 4.2.2 Ecological issues

The environment is always mentioned together with human health, cultural heritage, and economic activity when the FD describes the purpose of flood risk management and the adverse consequences of floods. The FD recognizes that floods damage the environment; however, the risk is considered insignificant if it threatens an unpopulated area with limited ecological value. As flood management is integrated into general water management, most ecological elements in the FD are indirectly included through the WFD and its environmental objectives. No particular participation of stakeholders representing nonhuman interests is mentioned in the FD but some specific information should be considered, such as the potential environmental pollution resulting from floods and the assessment of the effects of potential flood management measures on the environment. Moreover, the WFD recommends taking into account the vulnerability of aquatic ecosystems. In general, the strategies should reduce damage to the environment due to floods and promote environmental objectives, in line with the WFD and the Charter of Fundamental Rights of the European Union. In addition, flood risk management should consider giving more space to rivers and using some floodplains as natural flood retention areas. Although the FD does not mention responsibilities for ecological harm, the restoration of floodplains is suggested, and it is for example possible to use the European Union Solidarity Fund to help natural zones to return to pre‐disaster conditions. The WFD suggests the polluter‐pays principle for the restoration of aquatic environments.

#### 4.2.3 Spatial issues

The FD recognizes that different types of floods affect different places in the European Union, and that the causes and consequences of floods vary across geographical locations. Spatial aspects are strongly emphasized: Whereas each Member State is responsible for the flood risk management on its own territory, coordination must take place at the river basin level even if it is an international one. Therefore, collaboration with neighboring countries is essential, and transnational effects must be considered in cost and benefit analyses. Decision‐making should consider the particular needs and priorities of the specific geographical area at stake, and mapping risk assessments need to be done at the appropriate scale. Given the European solidarity principle, a Member State is not allowed to implement a measure that reduces flood risks on its own territory if it increases the risk of floods in another Member State, unless there is a specific agreement between them. In addition, a Member State facing an emergency can receive support and assistance from other Member States.

#### 4.2.4 Temporal issues

The management of flood risk should focus on prevention, protection, and preparedness, and as the FD respects the Charter of Fundamental Rights of the European Union, there are responsibilities and duties toward future generations (preamble). The FD requires the consideration of long‐term socioeconomic and natural developments, such as the impact of climate change on the occurrence and the impacts of floods. The FD does not directly include representatives of the interests of future generations in the decision‐making process, but the participation of children is indirectly encouraged through the Charter of Fundamental Rights of the European Union (article 24). Regular reviews and updates of flood risk management plans help distribute the effects over time. In addition, past flood events, their impacts, and the likelihood of similar events in the future guide strategy choices. Finally, according to the WFD, the selection of recovery measures needs an economic analysis of water services based on long‐term forecasts, and the measures taken to face exceptional circumstances such as floods should not compromise the recovery of water quality once the circumstances are over.

## 5 CONCLUDING DISCUSSION

To conclude, we discuss both the results from the documents analysis and the benefits of the risk justice framework. We also suggest some ideas for further research.

### 5.1 Similarities and differences between the SFDRR and the FD

The content analysis of the SFDRR and FD through the lens of our conceptual risk justice framework has underlined that many elements of these guidelines relate, even if only implicitly, to several forms and dimensions of justice. In particular, social and spatial dimensions of procedural and distributive justice are highlighted in the two documents, and both have a similar emphasis on the importance of prevention and preparedness rather than reactive actions. This aligns with increasing the well‐being of current and future generations by reducing risks, which is an essential aspect of sustainable development. Yet, we have identified some gaps in the SFDRR and the FD that may reduce the contribution of disaster and flood risk management to sustainable development. Notably, although ecological issues are considered, they are mostly focused on environmental protection for the purpose of human well‐being. Elements related to fairness among different nonhuman entities are scarce in the documents. In addition, the ecological and temporal aspects of procedural justice focus mostly on the consideration of information regarding impacts on future generations and ecosystems, and very little on the inclusion of participants representing their interests. For the temporal aspect, hearing the voices of children and youth is recommended; however, there is nothing on the interests of further generations. Corrective justice elements are also limited in both documents. In the SFDRR, some elements can be related to the restoration of the pre‐disaster situation, but no attribution of harm is explicit. In the case of flood risk management, the suggestion to apply a polluter‐pays principle for environmental injustices due to floods is indirect through the WFD and is not made explicit in the FD. These absences of direct statements may reflect the low priority given to some fairness aspects compared to others that are more explicit in the text. Concerning the limited attention given to corrective justice, a possible explanation involves the type of guidelines that are analyzed here. Both documents focus on the management of potential future risks rather than already existing harm, which would be the remit of corrective actions in risk management practice. Yet, we suggest that discussing justice issues proactively before harm occurs could support implementation of actions to correct it. Although the influence of international agreements on disaster risk reduction has been limited because of resistant sociopolitical structures (Raikes et al., 2022), we argue that discussing justice issues explicitly in international guidelines such as the SFDRR and the FD could be a first step toward an institutional change in the direction of sustainable development.

### 5.2 Benefits of the conceptual framework and further research

The risk justice framework presented here enables decision‐makers, researchers, evaluators, and all affected stakeholders to address justice issues explicitly and comprehensively in risk governance and management relating to different risk contexts. It connects and adds to other existing justice frameworks by enabling a systemic understanding of justice considerations in different risk management situations, thereby proactively addressing potential conflicts about what is perceived as fair processes and outcomes in order to eventually contribute to sustainable development. As our framework offers a new conceptual understanding, it promotes new ideas and encourages new ways of thinking, which is the benefit of conceptual research (MacInnis, 2011).

As shown in our application, using a risk justice framework to analyze international guidelines for disaster risk management enables us to identify gaps and omissions and hence question the contribution of disaster risk reduction to the well‐being of human populations, natural ecosystems, and future living entities. The risk justice framework has therefore been a useful retrospective analytical tool for pointing out what dimensions of justice are included in the guidelines, explicitly or implicitly, and what dimensions are not. By extension, this provides information on the potential contributions to (un)sustainable development practices. When fairness issues are not explicitly addressed, they may in practice be left open for the personal interpretation of the decision‐makers. Future international guidelines for disaster risk reduction could benefit from a discussion facilitated with the help of the risk justice framework presented in this article in order to explicitly point out the different forms of justice related to sustainable development. Doing so can help minimize potential conflicts associated with the implementation of disaster risk management strategies and make the choices of what should or should not be included to promote fairness more transparent.

Stakeholders willing to operationalize risk justice as a proactive decision‐making tool will have to determine justice principles that guide the answer to the question of “what is fair?,” and these principles are very much context‐dependent (de Goër de Herve, 2022). One of the advantages of the risk justice framework is that it can be used whatever justice principles apply in the specific context. Other studies could also test the framework on different risk management guidelines, applied to different kinds of risks, which are not necessarily disaster risks.

Thomalla et al. (2018) point out that disaster risk reduction actors often fail to consider various trade‐offs and that the current ways of building resilience are not always equipped to tackle issues of social inequity and injustice. We argue that the risk justice framework can facilitate discussions among practitioners and enable them to consciously address these issues. To transform the risk justice conceptual framework into a proactive decision support tool, further studies are required, especially research based on validity workshops with practitioners such as risk managers, to determine its applicability in real‐life processes of risk governance.

## CONFLICT OF INTEREST STATEMENT

The authors declare no conflict of interest.

## ORCID

Mathilde de Goër de Herve: 0000‐0002‐9667‐440X

Thomas Schinko: 0000‐0003‐1156‐7574

John Handmer: 0000‐0002‐6674‐7946

## Supporting information

Supporting Information

## Footnotes

1 The Charter of Fundamental Rights of the European Union was first published in 2000 and has been updated in 2012. In this analysis, we have used the updated version in spite of the fact that the FD was published in 2007.

## APPENDIX A

### A.1

This appendix presents the documents analyzed.

**Table:**

	Sendai Framework for Disaster Risk Reduction 2015–2030	EU Floods Directive
Purpose	Concise, focused, forward‐looking, and action‐oriented international Framework for disaster risk reduction following the Hyogo Framework for Action	“A ‘directive’ is a legislative act that sets out a goal that all EU countries must achieve. However, it is up to the individual countries to devise their own laws on how to reach these goals.” ^a^
Main focus	“The substantial reduction of disaster risk and losses in lives, livelihoods and health and in the economic, physical, social, cultural and environmental assets of persons, businesses, communities and countries.” p. 12	Prevention and mitigation of floods.
Year of publication	2015	2007
Source	United Nations	European Union

^a^ Definition available on https://european‐union.europa.eu/institutions‐law‐budget/law/types‐legislation_en (last access: November 2022)

## APPENDIX B

### B.1

This appendix details the list of codes used for the content analysis processed in NVIVO.

**Table:**

Name of the code	Description of the content	Examples
Distr_soc_risk	Elements related to social issues in the distribution of the risk itself	Who benefits from the risk? Directly, and indirectly? Who is exposed to the risk? Who is vulnerable to the risk?
Distr_soc_management	Elements related to social issues in the distribution of the management strategies	Who is targeted by the management strategy? Who implements it? Who pays for it? Who would benefit from a different strategy?
Distr_eco_risk	Elements related to ecological issues in the distribution of the risk itself	How are ecosystems impacted by the risk?
Distr_eco_management	Elements related to ecological issues in the distribution of the management strategies	How are ecosystems impacted by the management strategy? Is one type of ecosystem more impacted than another type?
Distr_spa_risk	Elements related to spatial issues in the distribution of the risk itself	Where does the risk take place? Are the ones benefiting from the risk and the ones at risk located in different areas?
Distr_spa_management	Elements related to spatial issues in the distribution of the management strategies	Does the management strategy impact different areas? How? Are the ones who benefit from the strategy located in a different place than the ones who carry the burdens of it?
Distr_temp_risk	Elements related to temporal issues in the distribution of the risk itself	When does the risk take place? Are there early warning systems? Are the ones creating the risk and the ones being at risk present at different points in time?
Distr_temp_management	Elements related to temporal issues in the distribution of the management strategies	What will be the impacts of the strategy on future generations? Are the ones benefiting from the strategy and the ones carrying the burdens of it in a different moment in time?
Corr_soc_harm	Elements related to social issues in the creation or the allocation of the harm	Who is responsible for the creation of harm? Who is affected (or potentially affected) by the harm?
Corr_soc_correction	Elements related to social issues in the actions to correct the harm	Who should correct the harm? How?
Corr_eco_harm	Elements related to ecological issues in the creation or the allocation of the harm	What ecological entities are harmed? For what purpose?
Corr_eco_correction	Elements related to ecological issues in the actions to correct the harm	How should ecosystems be restored? Who should be responsible for it? Who is financing and implementing the restorative actions?
Corr_spa_harm	Elements related to spatial issues in the creation or the allocation of the harm	Where is the harm done? Where are the ones responsible for the harm based?
Corr_spa_correction	Elements related to spatial issues in the actions to correct the harm	Where are the ones correcting the harm situated? Is the harm corrected where it takes place? At what spatial level are the corrective actions decided?
Corr_temp_harm	Elements related to temporal issues in the creation or the allocation of the harm	Is a given generation harming another one? Are the actions of today harming entities in the future?
Corr_temp_correction	Elements related to temporal issues in the actions to correct the harm	How should future harm be corrected? How is past harm corrected today?
Proc_soc_participation	Elements related to social issues in the involvement of stakeholders during the decision‐making process	Who should participate in the decision‐making process? How?
Proc_soc_info	Elements related to social issues in the information processed to make the decision	What information related to the effects of the risk management on different socioeconomic groups is considered?
Proc_eco_participation	Elements related to ecological issues in the involvement of stakeholders during the decision‐making process	Is there any representative of nonhumans in the decision‐making process? How are nonhumans represented?
Proc_eco_info	Elements related to ecological issues in the information processed to make the decision	How are the interests of nonhumans considered? How are the effects of risk management on nonhumans considered?
Proc_spa_participation	Elements related to spatial issues in the involvement of stakeholders during the decision‐making process	At what spatial level should the decisions be taken? Where are the decision‐makers situated?
Proc_spa_info	Elements related to spatial issues in the information processed to make the decision	How are the impacts on various geographical scales considered? Are the indirect impacts of risk management in other places taken into consideration?
Proc_temp_participation	Elements related to temporal issues in the involvement of stakeholders during the decision‐making process	Is there any representative of future generations in the decision‐making process? How are future generations represented? Should past generations be represented?
Proc_temp_info	Elements related to temporal issues in the information processed to make the decision	How are the interests of future stakeholders considered? How are considered the effects of risk management in the future? How are past risk management taken into account to inform future ones?

## References

[risa14157-bib-0001] Adger, W. N. , & Nelson, D. R. (2010). Fair decision making in a new climate of risk. In K. O'Brien , A. L. St. Clair , & B. Kristoffersen (Eds.), Climate change, ethics and human security (pp. 83–94). University of Cambridge Press. 10.1017/CBO9780511762475

[risa14157-bib-0002] Agyeman, J. (2013). Introducing just sustainabilities: Policy, planning, and practice. Zed Books.

[risa14157-bib-0003] Begg, C. (2018). Power, responsibility and justice: A review of local stakeholder participation in European flood risk management. Local Environment, 23(4), 383–397. 10.1080/13549839.2017.1422119

[risa14157-bib-0004] Bevere, L. , & Remondi, F. (2022). Natural catastrophes in 2021: The floodgates are open. Swiss Re Institute. https://www.swissre.com/dam/jcr:326182d5‐d433‐46b1‐af36‐06f2aedd9d9a/swiss‐re‐institute‐sigma‐natcat‐2022‐en.pdf

[risa14157-bib-0005] Busby, J. , & Sedmak, M. (2011). Practices and problems in the management of risk redistributions. Journal of Risk Research, 14(2), 259–279. 10.1080/13669877.2010.528561

[risa14157-bib-0006] Cambridge Dictionary . (n.d.‐a). Justice. In Cambridge Dictionary. Retrieved September 30, 2022, from https://dictionary.cambridge.org/dictionary/english/justice

[risa14157-bib-0007] Cambridge Dictionary . (n.d.‐b). Risk. In Cambridge Dictionary. Retrieved September 30, 2022, from https://dictionary.cambridge.org/dictionary/english/risk

[risa14157-bib-0008] Charter of Fundamental Rights of the European Union 2012/C 326/02, 2012 O. J. (C 326) 391. http://data.europa.eu/eli/treaty/char_2012/oj

[risa14157-bib-0009] CRED . (2022). 2021. Disasters in numbers: Extreme events defining our lives. https://reliefweb.int/report/world/2021‐disasters‐numbers

[risa14157-bib-0010] de Goër de Herve, M. (2021). Risk justice and the people, nature, place and time: A theoretical framework for fairness considerations in sustainable risk management. Poster presented at the Society for Risk Analysis‐European Conference 2021, Espoo, Finland (online). 10.13140/RG.2.2.20265.93289

[risa14157-bib-0011] de Goër de Herve, M. (2022). Fair strategies to tackle unfair risks? Justice considerations within flood risk management. International Journal of Disaster Risk Reduction, 69, 102745. 10.1016/j.ijdrr.2021.102745

[risa14157-bib-0012] Delforge, D. , Below, R. , & Speybroeck, N. (2022). Natural hazards & disasters: An overview of the first half of 2022. CRED Crunch Newsletter, (68). https://reliefweb.int/report/world/cred‐crunch‐newsletter‐issue‐no‐68‐september‐2022‐natural‐hazards‐disasters‐overview‐first‐half‐2022

[risa14157-bib-0013] Dietrich, M. (2021). Addressing inequal risk exposure in the development of automated vehicles. Ethics and Information Technology, 23(4), 727–738. 10.1007/s10676-021-09610-1

[risa14157-bib-0014] Directive 2000/60/EC of the European Parliament and the Council of 23 October 2000 establishing a framework for Community action in the field of water policy, 2000 O. J. (L 327) 1. http://data.europa.eu/eli/dir/2000/60/oj

[risa14157-bib-0015] Directive 2007/60/EC of the European Parliament and of the Council of 23 October 2007 on the assessment and management of flood risks (Text with EEA relevance), 2007 O. J. (L 288) 27. http://data.europa.eu/eli/dir/2007/60/oj

[risa14157-bib-0016] General Assembly Resolution , (Sep. 25, 2015). 70/1, Transforming Our World: The 2030 Agenda for Sustainable Development. https://www.un.org/en/development/desa/population/migration/generalassembly/docs/globalcompact/A_RES_70_1_E.pdf

[risa14157-bib-0017] Haimes, Y. Y. (2009). On the complex definition of risk: A systems‐based approach. Risk Analysis, 29(12), 1647–1654. 10.1111/j.1539-6924.2009.01310.x 19948003

[risa14157-bib-0018] Hermansson, H. (2010). Towards a fair procedure for risk management. Journal of Risk Research, 13(4), 501–515. 10.1080/13669870903305903

[risa14157-bib-0019] Hunjra, A. I. , Azam, M. , Bruna, M. G. , Verhoeven, P. , & Al‐Faryan, M. A. S. (2022). Sustainable development: The impact of political risk, macroeconomic policy uncertainty and ethnic conflict. International Review of Financial Analysis, 84, 102370. 10.1016/j.irfa.2022.102370

[risa14157-bib-0020] IPCC . (2012). Managing the risks of extreme events and disasters to advance climate change adaptation. Cambridge University Press. https://www.ipcc.ch/site/assets/uploads/2018/03/SREX_Full_Report‐1.pdf 10.1136/jech-2012-201045 22766781

[risa14157-bib-0021] ISO . (2018). *Risk management – Guidelines* (ISO Standard No. 31000:2018). ISO. https://www.iso.org/standard/65694.html

[risa14157-bib-0022] Izumi, T. , Shaw, R. , Ishiwatari, M. , Djalante, R. , Komino, T. , Sukhwani, V. , & Adu Gyamfi, B. (2020). 30 innovations linking Disaster Risk Reduction with Sustainable Development Goals. IRIDeS; Keio University; The University of Tokyo; UNU‐IAS; CWS Japan. https://www.preventionweb.net/files/70713_7071330innovationslinkingdrrwithsdg.pdf

[risa14157-bib-0023] Jaccard, J. , & Jacoby, J. (2020). Theory construction and model‐building skills: A practical guide for social scientists (2nd ed.). Guilford Press.

[risa14157-bib-0024] Johannesson, P. , Zhemchugova, H. , & Hanger‐Kopp, S. (2022). An ontological analysis of justice. In Proceedings of the 16th International Workshop on Value Modelling and Business Ontologies (VMBO 2022), held in conjunction with the 34th International Conference on Advanced Information Systems Engineering (CAiSE 2022), June 06–10, 2022, Leuven, Belgium. https://ceur‐ws.org/Vol‐3155/paper1.pdf

[risa14157-bib-0025] Kaufmann, M. , Priest, S. , Hudson, P. , Löschner, L. , Raška, P. , Schindelegger, A. , Slavíková, L. , Stričević, R. , & Vleesenbeek, T. (2021). Win–win for everyone? Reflecting on nature‐based solutions for flood risk management from an environmental justice perspective. In C. S. S. Ferreira , Z. Kalantari , T. Hartmann , & P. Pereira (Eds.), The Handbook of Environmental Chemistry: Vol. 107. Nature‐based solutions for flood mitigation: Environmental and socio‐economic aspects (pp. 399–423). Springer. 10.1007/978-3-030-77505-6

[risa14157-bib-0026] Lazar, S. R. M. (2008). Corrective justice and the possibility of rectification. Ethical Theory and Moral Practice, 11(4), 355–368. 10.1007/s10677-008-9108-8

[risa14157-bib-0027] MacInnis, D. J. (2011). A framework for conceptual contributions in marketing. Journal of Marketing, 75(4), 136–154. 10.1509/jmkg.75.4.136

[risa14157-bib-0028] MacMillian Dictionary . (n.d.‐a). Justice. In MacMillian Dictionary. Retrieved September 30, 2022, from https://www.macmillandictionary.com/dictionary/british/justice

[risa14157-bib-0029] MacMillian Dictionary . (n.d.‐b). Risk. In MacMillian Dictionary. Retrieved September 30, 2022, from https://www.macmillandictionary.com/dictionary/british/risk_1

[risa14157-bib-0030] Martinuzzi, A. , & Meyer, W. (2016). Evaluating sustainable development in a global society. In R. Stockmann & W. Meyer (Eds.), The future of evaluation: Global trends, new challenges, shared perspectives (pp. 81–94). Palgrave Macmillan. 10.1057/9781137376374

[risa14157-bib-0031] Mullen, E. , & Okimoto, T. (2015). Compensatory justice. In R. S. Cropanzano & M. L. Ambrose (Eds.), The Oxford handbook of justice in the workplace (pp. 477–496). Oxford University Press. 10.1093/oxfordhb/9780199981410.013.23

[risa14157-bib-0032] O'Hare, P. , & White, I. (2018). Beyond ‘just’ flood risk management: The potential for—and limits to—alleviating flood disadvantage. Regional Environmental Change, 18(2), 385–396. 10.1007/s10113-017-1216-3

[risa14157-bib-0033] Oxford Learner's Dictionaries . (n.d.). Justice. In Oxford Learner's Dictionaries. Retrieved September 30, 2022, from https://www.oxfordlearnersdictionaries.com/definition/english/justice?q=justice

[risa14157-bib-0034] Pahl‐Wostl, C. , Becker, G. , Knieper, C. , & Sendzimir, J. (2013). How multilevel societal learning processes facilitate transformative change: A comparative case study analysis on flood management. Ecology and Society, 18(4), 58. 10.5751/ES-05779-180458

[risa14157-bib-0035] Perry, R. (2010). The third form of justice. Canadian Journal of Law and Jurisprudence, 23(1), 233–247. 10.1017/S0841820900004884

[risa14157-bib-0036] Raikes, J. , Smith, T. F. , Baldwin, C. , & Henstra, D. (2022). The influence of international agreements on disaster risk reduction. International Journal of Disaster Risk Reduction, 76, 102999. 10.1016/j.ijdrr.2022.102999

[risa14157-bib-0037] Ruggerio, C. A. (2021). Sustainability and sustainable development: A review of principles and definitions. Science of the Total Environment, 786, 147481. 10.1016/j.scitotenv.2021.147481 33965820

[risa14157-bib-0038] Šakić Trogrlić, R. , Donovan, A. , & Malamud, B. D. (2022). Invited perspectives: Views of 350 natural hazard community members on key challenges in natural hazards research and the Sustainable Development Goals. Natural Hazards and Earth System Sciences, 22(8), 2771–2790. 10.5194/nhess-22-2771-2022

[risa14157-bib-0039] Schlosberg, D. (2007). Defining environmental justice: Theories, movements, and nature. Oxford University Press. 10.1093/acprof:oso/9780199286294.001.0001

[risa14157-bib-0040] Spurlock, C. A. , Elmallah, S. , & Reames, T. G. (2022). Equitable deep decarbonization: A framework to facilitate energy justice‐based multidisciplinary modeling. Energy Research and Social Science, 92, 102808. 10.1016/j.erss.2022.102808

[risa14157-bib-0041] SRA . (2018). Society for risk analysis glossary. SRA. https://www.sra.org/wp‐content/uploads/2020/04/SRA‐Glossary‐FINAL.pdf

[risa14157-bib-0042] Thaler, T. (2021). Just retreat—How different countries deal with it: Examples from Austria and England. Journal of Environmental Studies and Sciences, 11(3), 412–419. 10.1007/s13412-021-00694-1

[risa14157-bib-0043] Thomalla, F. , Boyland, M. , Johnson, K. , Ensor, J. , Tuhkanen, H. , Swartling, Å. G. , Han, G. , Forrester, J. , & Wahl, D. (2018). Transforming development and disaster risk. Sustainability, 10(5), 1458. 10.3390/su10051458

[risa14157-bib-0044] United Nations . (2015). Sendai framework for disaster risk reduction 2015 ‐ 2030. UNISDR. https://www.preventionweb.net/files/43291_sendaiframeworkfordrren.pdf

[risa14157-bib-0045] Wallimann‐Helmer, I. , Meyer, L. , Mintz‐Woo, K. , Schinko, T. , & Serdeczny, O. (2019). The ethical challenges in the context of climate loss and damage. In R. Mechler , L. M. Bouwer , T. Schinko , S. Surminski , & J. Linnerooth‐Bayer (Eds.), Loss and damage from climate change: Concepts, methods and policy options (pp. 39–62). Springer. 10.1007/978-3-319-72026-5

[risa14157-bib-0046] Weinrib, E. J. (2002). Corrective justice in a nutshell. The University of Toronto Law Journal, 52(4), 349–356. 10.2307/825933

[risa14157-bib-0047] World Commission on Environment and Development . (1987). Our Common Future. Oxford University Press.

