# Supplementary material for: Risk justice: Boosting the contribution of risk management to sustainable development
Source: Risk Anal. 2023 May 21;45(11):3452–66. doi: 10.1111/risa.14157 (PMC12663896; doi:10.1111/risa.14157)
Supplement: Supplementary file 1 — Supporting Information [file RISA-45-3452-s001.docx]

**Risk Justice: Boosting the contribution of risk management to sustainable development**

**Mathilde de Goër de Herve, Thomas Schinko, John Handmer**

**SUPPLEMENTARY MATERIAL**

**A1 – Detailed description of the results for the Sendai Framework for Disaster Risk Reduction 2015-2030**

**4.1. Sendai Framework for Disaster Risk Reduction 2015-2030 (SFDRR)**

The SFDRR includes several elements of procedural and distributive justice. However, there is very limited consideration of corrective justice, which is almost absent apart from aspects of insurance and international finance for post-disaster recovery, and discussions of ex-post resettlement and migration policies. An overview of the issues contained in the SFDRR using the risk justice framework is presented here. The results are presented in terms of sustainability-related dimensions (i.e. social, ecological, spatial, and temporal justice) rather than the meta dimensions of justice (i.e., procedural, distributive, and corrective justice) to focus on how the SFDRR engages with sustainable development issues from a justice perspective. In summary, the SFDRR has a strong focus on social and spatial sustainability dimensions with many explicit statements in these contexts. There is less emphasis on the temporal dimension, and the ecological dimension is relatively under-represented in the SFDRR.

*4.1.1. Social issues*

Concerning social issues, the SFDRR argues that the management of disaster risks should be people-centered and preventive and promotes, in terms of distributional justice, the empowerment and support of people disproportionately affected by disasters (e.g., women, the elderly, migrants, indigenous groups). The main strategy is to invest in the resilience of people and communities according to their respective vulnerabilities and needs in order to reduce potential losses and damages. The measures should integrate different institutional key dimensions with a focus on tackling root causes of disaster risks by investing in poverty and hunger reduction, as well as in educational, health, and telecommunication infrastructure. According to the SFDRR, both the affected people and the host communities should be supported in the aftermath of a disaster. Clear tasks and responsibilities for risk management must be assigned to relevant stakeholders, which also involves the allocation of needed resources.

Corrective justice aspects in the social dimension refer to the need for accountability in relation to disaster risk creation at all levels. The SFDRR also suggests the promotion of mechanisms for international disaster risk transfer and insurance, risk-sharing and retention and financial protection, which can be interpreted as corrective measures.

The SFDRR highlights procedural justice aspects in many areas and particularly encourages all-society engagement in the decision-making process, with special attention to giving voice to people disproportionally affected by disasters, since the SFDRR recognizes that some are indeed disproportionally affected. The process should be empowering and inclusive with accessible and non-discriminatory participation. An example given in the document is that people with life-threatening and chronic diseases should be part of the design and implementation of the management of the risk threatening them. Collaborative decision-making should include all stakeholders in society, public and private, at different institutional levels and in different sectors, including for instance the scientific and technological communities. The SFDRR also underlines the importance of the commitment and involvement of political leadership at all levels. The decision-making should be based on various types of knowledge, including scientific, but also traditional and indigenous knowledge, as well as knowledge coming from the experiences of women, migrants, and experts. The needs of different categories of users should be taken into consideration, and this is facilitated by the collection of disaggregated data by, for instance, sex, age, and disability. All non-sensitive data should be shared among the stakeholders, and the choice of a strategy should be based on information about the understanding of the risk and its dimensions, as well as the impacts of the potential strategies on the economic, social, health, education, environmental sectors, and cultural heritage. Information in the interest of sustainable social and economic development must also be included.

*4.1.2. Ecological issues*

Ecological elements are less in focus in the SFDRR compared to social aspects, yet they are mentioned in several ways. No representative for non-human interests is suggested as a participant in the decision-making process, but choices should be made considering the vulnerability, capacity, and exposure of the environment as well as the effects of disasters on ecosystems and environmental heritage. What is called ‘environmental challenges’ in the document must be considered when implementing measures. The management of disaster risks aims, among other goals, at protecting environmental assets and ecosystems, with a strong focus on investing in environmental resilience. This goes hand in hand with environmental and resource management and necessitates collaboration with other mechanisms such as promoting biodiversity. Special attention is given to the protection of livestock, working animals, and seeds. It is possible to use ecosystem-based approaches to manage disaster risks, and when encouraging human settlements in areas considered safe, the ecosystem functions that help reduce risks need to be preserved. We can therefore note that ecosystems are considered mostly for the benefit of human needs, and so this aspect of the SFDRR touches upon the question of fairness between human and non-human entities more than fairness between different non-human entities. Another missing element is corrective measures for ecological purposes.

*4.1.3. Spatial issues*

There is a strong emphasis in the SFDRR on spatial issues, since the guidelines for each priority are categorized for different spatial levels. The SFDRR has a dedicated section on “International cooperation and global partnership”, and focuses in particular on procedural and distributive international justice aspects. Participation in the decision-making process occurs notably through the cooperation between various spatial levels (international, regional, subregional, and transboundary levels as well as local and national levels in each State) and international collaboration mechanisms. From a distributive justice perspective, there is an emphasis on help coming from the ‘developed’ countries to the ‘developing’ ones (terms used in the SFDRR), as well as ‘South-South’ and triangular cooperation. When implementing this kind of collaboration that ranges from financial and technical assistance to capacity building and technology transfers, the choice of strategies should be based on the needs and priorities identified by the beneficiaries themselves. In general, local characteristics of disaster risks should be taken into consideration during the decision-making process and information gathered by geospatial technologies can help. The SFDRR acknowledges that some countries face specific challenges because of higher vulnerability and risk levels and are therefore disproportionally affected by higher mortality and economic losses. Finally, the SFDRR encourages the adoption of policies and programs addressing disaster-induced human mobility to strengthen the resilience of affected people and that of host communities, and it also suggests consideration of the relocation of public facilities and infrastructure to areas outside the risk range in the post-disaster reconstruction process. These two last aspects can be interpreted as corrective actions.

*4.1.4. Temporal issues*

Temporal aspects are included in the SFDRR, and the participation and leadership of children and youth, representing the interests of the currently young and future generations, are strongly encouraged as they are agents of change, as well as the participation of older persons because they accumulate knowledge, skills, and wisdom over the years. Moreover, since climate change is considered a driver of disaster risks, information about it should be included, for instance through climate scenarios. In a more general sense, factors and scenarios for disaster risks in the medium and long term should be developed. In making decisions, it is also important to learn from past programs and disaster reviews. In addition to climate change impacts on the frequency and intensity of some disaster risks, the fact that exposure is increasing faster than the decrease in vulnerability affects the consequences of disaster risks in the short, medium, and long term. According to the SFDRR, the management of disaster risks should prevent future losses by focusing on prevention and preparedness as it is the most cost-effective approach. This includes the emphasis on investment in resilience through tackling root causes of disaster risks. In addition, new infrastructure should be built in a manner that is resilient to the forecasted disasters, and when a disaster strikes, ‘building back better’ is essential to prevent the creation of new risks and reduce existing ones. During the recovery phase, the capacities should be developed to reduce risks in the short, medium, and long term. However, there is no specific mention of corrective actions with a temporal dimension.

**A2a – Summary table presenting elements related to justice in the Sendai Framework for Disaster Risk Reduction 2015-2030**

|  | **Procedural** | **Distributive** | **Corrective** |
| --- | --- | --- | --- |
| **Social** | **Participation**:  The SFDRR has a separate section for the role of stakeholders, which shows the importance of procedural issues.  All-society engagement and partnership with empowerment and inclusive, accessible, and non-discriminatory participation. Special attention given to people disproportionally affected by disasters.  Full and meaningful participation of relevant stakeholders (from governments to private actors, as well as civil societies, academia, and individuals) for both the design and implementation of disaster risk reduction at appropriate levels.  Promotion of women (their participation is critical to effectively manage risks) and youth leadership (children and youth as agents of change). Emphasis as well on the participation of persons with disabilities (to tailor plans to their specific requirements) and life-threatening and chronic diseases (to tailor strategies to their needs), poor people, migrants (for their contribution to the resilience of communities and societies, and their knowledge, skills, and capacities to be useful), indigenous people (for their experience and traditional knowledge), volunteers, community of practitioners, and older persons (for their years of knowledge, skills, and wisdom).  Coordination across diverse relevant institutions, sectors and levels, including cooperation between public and private stakeholders (including affected communities and business).  Involvement of the scientific and technological community.  Involvement of community-based organizations and nongovernmental organizations to enhance collaboration at the local level and disseminate information. Empowerment of local authorities and local communities through regulatory and financial means.  Importance of the public and community consultations.  Commitment and involvement of political leadership at all levels to implement the SFDRR.  Contribution of media to raising public awareness and understanding.  Support and review of the SFDRR by the United Nations.  Overall responsibility for reducing disaster risk lied with the States, but shared responsibility between governments and stakeholders to take action (in particular non-State stakeholders as enablers). Assignment of clear roles and tasks to community representatives through relevant legal frameworks.  **Information:**  Inclusion of scientific knowledge, traditional knowledge, indigenous knowledge, experience knowledge from migrants, women knowledge, evidence-based knowledge from experts.  Use of disaggregated data, including by sex, age, and disability. Inclusion of a gender, age, disability and cultural perspective in all policies and practices.  Considering disaster risk in all its dimensions of vulnerability, capacity, exposure of persons, communities, countries, and assets, hazard characteristics, and the environment.  Considering the impacts on economic, social, health, education, environmental and cultural heritage.  Importance of taking into account the needs of different categories of users.  Use of post-disaster reviews for learning.  Use of guidelines and follow-up tools informed by demographic changes.  Inclusion of information in the interest of sustainable social and economic development.  Consideration of national laws and regulation.  Sharing among stakeholders and countries information, disaggregated data, lessons learned, best practices, and all non-sensitive data.  Encouraging the availability of copyrighted and patented material, through negotiated concessions if needed. | **Risk and materialized impacts:**  Some people are disproportionally affected by disasters, such as women, children, and people in vulnerable situations.  Recurring small-scale and slow-onset disasters affect communities, households, and small and medium enterprises in particular.  **Risk management:**  Focus on people-centered and preventive disaster risk reduction (including actions dedicated to tackling underlying disaster risk drivers and root causes such as poverty, inequality, hunger).  Empowering and assisting people disproportionately affected by disasters.  Focus health and livelihoods to build resilience and reduce losses and damages.  Strengthening social-safety nets mechanisms, such as for health, education, alimentation. Investment in economic, social, health, cultural, and educational resilience of persons, communities, countries, and the environment.  Promotion of resilience: of workplaces, national health systems, business, critical infrastructures (such as water transportation, telecommunication, hospitals, educational facilities). Resilience as a driver of innovation, growth, and job creation.  Promotion of integrated measures: economic, structural, legal, social, health, cultural, educational, environmental, technological, political, and institutional.  Promotion of a culture of disaster prevention resilience and responsible citizenship.  Promotion of human rights through disaster risk reduction, including the right to development.  Strengthening public education and awareness in disaster risk reduction, taking into account the specific audiences and their needs.  Complying with existing safety-enhancing provisions of sectoral laws and regulations, and integration of disaster risk management in all relevant sectors such as land-use and tourism industry.  Allocation of necessary resources to relevant stakeholders at all levels.  Promotion of risk transfer mechanisms such as insurance for both public and private societies (that, among others, reduce the financial impact of disasters in governments and societies).  Protection of sites of cultural, historical, and religious interest. Protection of livelihoods.  Investment in resilience of both the affected people and host communities.  Promotion of training to prepare.  Investment in people-centered, multi-hazard, multisectoral, simple and low-cost forecasting and early warning systems that are tailored to the needs of users.  Support to public service and voluntary workers for relief assistance and post-disaster actions.  Psychosocial support for those in need after a disaster.  Promotion of gender equitable and universally accessible response, recovery, rehabilitation, and reconstruction. | **Harmer/Harmed:**  Need for accountability for disaster risk creation at all levels.  **Corrective actions:**  Promotion of disaster risk transfers and insurance, risk-sharing, and retention and financial protection, with various stakeholders such as international community and financial institutions, business, and others.  Promotion of recovery, rehabilitation, and reconstruction approaches that are gender equitable and universally accessible. |

|  | **Procedural** | **Distributive** | **Corrective** |
| --- | --- | --- | --- |
| **Ecological** | **Participation:**  NOT INCLUDED  **Information:**  Understanding of the vulnerability, capacity, and exposure of the environment.  Consideration of effects on ecosystems and impacts of disasters on environmental heritage.  Considerations for environmental challenges in disaster risk reduction.  Use of guidelines and follow-up tools informed by environmental changes. | **Risk and materialized impacts:**  Environmental impacts resulting from new risks and a steady rise in disaster-related losses.  **Risk management:**  Protection of environmental assets and ecosystems and strengthening their resilience.  Investment in the resilience of the environment.  Implementation of environmental measures together with others to prevent new and reduce existing disaster risks.  Complying with environmental and resource management to ensure a focus on disaster risk management. Strengthening the sustainable use and management of ecosystems, and implementing integrated environmental and natural resource management approaches into disaster risk reduction.  Collaboration for coherent instruments and tools relevant for, among others, climate change, biodiversity, sustainable development and the environment.  Use of ecosystem-based approaches.  Inclusion of disaster risk management into rural development planning and management of, for example, mountains, rivers, coastal flood plain areas, drylands, wetland.  Identification of areas safe for human settlement and at the same time preserving ecosystem functions that help to reduce risks.  Protection productive assets and livelihoods, including livestock, working animals, tools, and seeds.  Financing environmentally sound technology. | **Harmer/Harmed:**  NOT INCLUDED  **Corrective actions:**  NOT INCLUDED |

|  | **Procedural** | **Distributive** | **Corrective** |
| --- | --- | --- | --- |
| **Spatial** | **Participation:**  Inclusion of all nations.  Involvement of national, regional, and global levels to strengthen good governance.  Cooperation between international, regional, subregional, and transboundary levels, as well as national and local levels involved in each State.  The SFDRR has a dedicated section for ‘international cooperation and global partnership’.  Importance of international, regional, subregional, transboundary and bilateral cooperation to prevent and reduce disaster risk, which is the primary responsibility of each State.  Focus on international cooperation between ‘developed’ and ‘developing’ countries, and between States and international organizations. Importance of ‘North-South’, ‘South-South’, and triangular cooperation.  Important role of regional intergovernmental organizations for regional platforms for disaster risk reduction.  Bilateral, regional, and multilateral collaborative arrangements (including United Nations) to enhance information sharing and access of States, including ‘developing’ countries, to knowledge, science, inclusive innovation, environmentally sound technology, and finance.  Encouragement of active engagement for awareness rising campaigns of public and private stakeholders at the local, national, regional, and global levels.  Necessity to engage with local authorities and communities.  **Information:**  Understanding and consideration for local characteristics of disaster risks.  Importance of location-based information, including risk maps.  Use of geospatial technologies for observations, such as GIS.  Necessity to base international cooperation on the needs and priorities identified by the beneficiary countries.  Promotion of information, experience, and non-sensitive data through international cooperation.  Consideration of respective capacities and capabilities, in line with national laws and regulation. | **Risk and materialized impacts:**  Recognition that new risks and the rise in disaster-related losses are created by exposure, in particular at the local and community levels.  The drivers of disaster risks may be local, national, regional in scope.  ‘Developing’ countries face disproportionately higher mortality and economic losses from disasters.  Some countries face specific challenges because of higher vulnerabilities and risk levels that exceed their capacities to respond and recover such as ‘the least developed’ countries, small island ‘developing’ States, landlocked ‘developing’ countries, African countries, and middle-income countries.  Some countries are disaster-prone because of specific characteristics such as archipelagic countries and countries with extensive coastlines.  Climate change exacerbates the effects of disasters for instance in some small island ‘developing’ States that are disproportionally affected and therefore their progress towards sustainable development are impeded.  **Risk management:**  Necessity of disaster risk management to take place at all levels. Focusing actions at local, national, regional, and global levels. Strengthening good governance at national, regional, and global levels.  Strengthening modalities for international cooperation, with for instance funding mechanisms for international assistance, capacity-building, financial and technical assistance, and technology transfers.  Importance of transboundary cooperation to implement ecosystem-based approaches with regard to shared resources. Importance of financing environmentally sound technology from a variety of international sources.  Promotion of international voluntary mechanisms for monitoring and assessment of risks.  Creation of common information systems (such as the International Recovery Platform) and exchange of good practices to address common and transboundary disaster risks.  Focus on international cooperation and genuine and durable partnerships at the regional and international levels to help the most disaster-prone countries. Special attention to disaster-prone countries with specific characteristics such as archipelagic countries and countries with extensive coastlines.  Focus on adequate, sustainable, and timely resources through continued international support towards ‘developing’ countries. Consideration of the SFDRR priorities by international financial institutions (such as the World Bank) for providing financial support and loans for integrated disaster risk reduction for ‘developing’ countries.  Importance of capacity development in disproportionally affected countries.  Importance, especially for ‘developing’ countries of concerted international cooperation, of enabling international environment, and means of implementation to develop knowledge, capacities, and motivation for disaster risk reduction in the context of increasing global interdependence.  Need for the fulfillment of respective commitments of official development assistance by ‘developed’ countries and need for sustainable international cooperation. Need for international efforts toward hunger and poverty eradication. Incorporation of disaster risk reduction measures into multilateral and bilateral development assistance programs within and across all sectors.  Need to strengthen the overall capacity of the United Nations system to assist ‘developing’ countries by providing adequate resources.  Collaboration for the coherence of instruments and tools across the global and regional mechanisms and coordination for preparation in case of disaster situations exceeding national coping capacities.  Sharing response capacities and resources during and after disasters at the regional level.  Implementation of appropriate strategies for local needs.  Investment in the resilience of persons, communities, and countries.  Consideration for relocation of human settlements when they are in disaster-prone areas. | **Harmed/Harmer:**  Need for accountability for disaster risk creation at all levels.  **Corrective actions:**  During the reconstruction process, consideration for relocation of human settlement, public facilities, and infrastructures outside the risk range (to be done in consultation with the people concerned).  Implementation of policies and programs to address human mobility due to disaster for strengthening the resilience of both the affected people and the host communities. |

|  | **Procedural** | **Distributive** | **Corrective** |
| --- | --- | --- | --- |
| **Temporal** | **Participation:**  Participation of children and youth, because they are agents of change, as well as older persons, because they have years of knowledge, skills, and wisdom. Promotion of youth leadership.  **Information:**  Consideration of climate change as a driver of disaster risk and therefore climate development through e.g. climate change scenarios.  Researching factors and scenarios for disaster risks in the medium and long term.  Learning from the recovery and reconstruction programs since the adoption of the previous framework (Hyogo Framework for Action). | **Risk and materialized impacts:**  Increasing frequency and intensity of some disasters because of climate change.  Impacts in the short, medium, and long term due to a faster increase in exposure than the decrease in vulnerability.  **Risk management:**  Necessity to invest today to reduce risk in the future (2030) for all the seven global targets.  Reduction of disaster risk as a cost-effective investment in preventing future losses: focus on prevention and preparedness, including actions on the risk drivers, such as poverty, inequality, climate change, etc.  Incorporation of disaster risk management into adaptation to climate change.  Importance of building back better to prevent the creation of new risks and reduce existing ones. Developing capacities to reduce risks in the short, medium, and long-term during the recovery phase.  Urgent and critical need to reduce disaster risks and strengthen resilience.  Focus on addressing existing challenges and preparing for future ones.  Investment in long-term solution-driven research.  Implementation of disaster risk reduction strategies across different timescales, to prevent the creation of risk, reduce existing one, and strengthen resilience.  Importance of regular follow up and reviewing plans.  Need to build infrastructures resilient to forecasted disasters.  To adopt and implement national and local disaster risk reduction strategies and plans, across different timescales, with targets, indicators and time frames, aimed at preventing the creation of risk, the reduction of existing risk and the strengthening of economic, social, health and environmental resilience. | **Harmed/Harmer:**  NOT INCLUDED  **Corrective actions:**  NOT INCLUDED |

**A2b – Examples of distributive justice elements present in the Sendai Framework for Disaster Risk Reduction 2015-2030**

| **Same place, same time** |  | **Different places, same time** | **Different places, different times** | **Same place, different times** |  |
| --- | --- | --- | --- | --- | --- |
| Priority to assist disproportionally affected people;  Poverty reduction, emphasis on health and education access | **SOCIAL ISSUES** | Disaster risk strategies at the local, national, regional, and global levels;  Assistance of ‘developing countries’ capacities by ‘developed countries’ through international cooperation | Governance in different timescales with established time frames for both national and local disaster risk reduction strategies and plans | Build Back Better;  Long-term investments in innovation and technology development | **Between humans** |
| Importance of managing natural assets and ecosystems to reduce risks for humans |  | Effects of disaster risks on ecosystems at different spatial scales | Coherence of disaster risk management with climate change and biodiversity actions | Address gaps, obstacles, interdependencies and social, educational and environmental challenges | **Between humans and non-humans** |
| Focus on working animals, livestock and seeds | **ECOLOGICAL ISSUES** | Not included | Not included | Investment in environmental resilience | **Between non-human entities** |
|  |  | **SPATIAL ISSUES** |  | **TEMPORAL ISSUES** |  |

**B1 – Detailed description of the results for the European Floods Directive**

**4.2. European Floods Directive (FD)**

The FD contains elements addressing all four sustainability dimensions and all three meta-level aspects of risk justice: procedural, distributive, and corrective. Most corrective justice elements as well as topics related to ecological sustainability are indirectly derived from another European directive to which the FD often refers, namely the Water Framework Directive (WFD). The WFD is older than the FD, but it does not directly aim at managing flood events even though it considers that floods increase the risk of spreading water pollution. Another important document that the FD refers to, and which indirectly adds substantially to the social sustainability dimension, is the Charter of Fundamental Rights of the European Union, which was proclaimed in 2000 and updated in 2012. The FD mentions the contribution of flood management to sustainable practices, through environmental protection, sustainable land use practices, and sustainable human activities. A detailed analysis of the FD elements from a risk justice perspective is presented here, including relevant elements present in those two other documents.

*4.2.1. Social issues*

Social perspectives, particularly in the context of procedural justice, are broadly included in the FD which suggests an active involvement of all interested parties in the production, review, and updating of management plans notably through public information and consultation. When appropriate, this participation can be coordinated together with the stakeholders involved in the WFD. When making decisions, the costs and benefits of the strategies must be considered, with for instance information on the number of inhabitants and the type of economic activity in the potentially affected area. Best practice cases and best available technological options are also relevant information according to the FD, and activities that increase flood risks should be assessed. Management plans should be adapted as science and technology progress. The management measures should prevent and reduce damage to human health, the environment, cultural heritage, and economic activity, and if possible reduce the likelihood of flooding. For distributive justice, the FD argues that there must be a “fair sharing of responsibilities” (p. 28) for measures jointly decided for the common benefit of the Community, in light of the solidarity principle. Since the FD respects the Charter of Fundamental Rights of the European Union, it must consider the right to life for everyone (article 2), the right to property (article 17), the prohibition of discrimination based on grounds such as sex, race, color, ethnic or social origin, genetic features, language, religion or belief, political or any other opinion, membership of a national minority, property, birth, disability, age or sexual orientation (article 21), gender equality (article 23) and the right to work in fair and healthy conditions (article 31). In addition, based on the WFD, the most stringent objective should usually apply in the case of multi-purpose use of bodies of water for different forms of sustainable human activities. Some exemptions from the environmental objectives of the WFD may be granted in exceptional circumstances, based on criteria given in the WFD. Finally, the FD recognizes that some human activities and climate change contribute to the creation of adverse impacts and harm from flood events. Addressing corrective justice issues, in case of a disaster event, the European Solidarity Fund can grant rapid financial assistance to help people to return to conditions as normal as possible. In general, corrective measures should be based on the polluter-pays principle according to the WFD.

*4.2.2. Ecological issues*

Elements related to the ecological dimension of risk justice in the FD are included mostly through the WFD. Yet, the environment is always mentioned together with human health, cultural heritage, and economic activity when the FD describes the purpose of flood risk management and the adverse consequences of floods. While the FD recognizes that floods damage the environment, the risk is considered as not significant if they threaten an unpopulated area with limited ecological value. Since flood management should be integrated into water management in general, the environmental objectives of the WFD must be applied when managing flood risks. This includes potential exceptions based on specific conditions described in the WFD.

In terms of procedural justice, no participation of representatives of non-human interests during the decision-making process is mentioned in the FD, yet some information should be considered, such as the potential sources of environmental pollution as a consequence of floods. The assessment of the effects of potential flood management measures on the environment should be included as well during the decision-making process. For an effective and coherent water policy, the WFD recommends taking into account the vulnerability of aquatic ecosystems. In terms of distribution of the management measures, the strategies should reduce damage to the environment due to floods and promote environmental objectives, in line with the WFD and the Charter of Fundamental Rights of the European Union. In addition, the management of flood risks should consider giving more space to rivers and using some floodplains as natural retention areas. In terms of corrective justice, there is no element in the FD informing the responsibilities for the harm caused, but the restoration of floodplains is suggested, and it is for instance possible to use the European Union Solidarity Fund to help natural zones to return to conditions as normal as possible after a flood disaster. Based on the WFD, the polluter-pays principle to recover costs associated with negative impacts on the aquatic environment is suggested.

*4.2.3. Spatial issues*

Spatial aspects are strongly emphasized in the FD since it is a legally binding agreement between the Member States of the European Union. In terms of distributive justice, each Member State is responsible for flood risk management on its own territory, and yet, from a procedural justice perspective, coordination must take place at the river basin level, both when it is entirely on the national territory, and when it is an international river basin. Therefore, collaboration with different neighboring countries is essential. In addition, if a Member State identifies an issue that it cannot resolve by itself, other Member States may advise on it. To take decisions on how to manage floods, the particular needs and priorities of specific areas should be considered, with the inclusion of local and regional circumstances into the plans. The assessment maps need to be created at the appropriate scale for the decisions, and the transnational effects of floods are to be included in the analysis of costs and benefits. Member States should exchange information, especially for international river basins. It is recognized by the FD that different types of floods affect different places in the European Union, and that the causes and consequences of floods vary across countries and regions. The most effective measures therefore should be taken at the river basin level, and all actions should consider the solidarity principle. In particular, it is not allowed for a Member State to implement a measure with the goal of reducing flood risks on its territory if this same measure increases the risk of floods in another Member State, either downstream or upstream, unless there is a specific agreement between all the concerned States. Another illustration of the solidarity principle is that in case of major emergencies, a Member State can receive support and assistance from the other Member States. From a corrective justice angle, there are no responsibilities established for harm, yet the WFD suggests considering the geographic and climatic conditions of the regions affected when developing and implementing recovery measures, which relate to the spatial dimension of corrective justice.

*4.2.4. Temporal issues*

Finally, some elements in the FD can be associated with the temporal dimension of risk justice, mostly through the attention given to long-term socioeconomic and natural developments, and in particular the impact of climate change on the occurrence of floods. The FD recognizes the increasing likelihood and adverse impacts of flood events over time due to climate change. In addition, past flood events and their impacts as well as the likelihood of similar events in the future should be considered when choosing a strategy. Despite no specific representative for the interests of future generations being directly mentioned for participation in the decision-making process, the Charter of Fundamental Rights stipulates that children should express their views freely, which should be taken into consideration on matters that concern them (article 24). The management of flood risk should focus on prevention, protection, and preparedness, and since the FD respects the Charter of Fundamental Rights of the European Union, there are responsibilities and duties towards future generations (preamble). Regular reviews and updates of flood risk management plans can help to distribute the effects over time. Concerning corrective justice, the choice of recovery measures should be informed by an economic analysis of water services based on long-term forecasts according to the WFD. In addition, the measures taken to face exceptional circumstances such as floods should not compromise the recovery of the quality of the bodies of water once the circumstances are over.

**B2 – Summary table presenting elements related to justice in the European Floods Directive**

|  | **Procedural** | **Distributive** | **Corrective** |
| --- | --- | --- | --- |
| **Social** | **Participation:**  Active involvement of all interested parties in the production, review, and updating of management plans via notably public information and consultation measures.  Coordination with the participants to the WFD if appropriate.   \| Respect of the Charter of Fundamental Rights of the European Union: integration of persons with disabilities (article 26); right to good administration (article 41). \| \| --- \|   **Information:**  Consideration for costs and benefits in the plans.  Assessment of activities that increase flood risks.  Use best practices cases and best available technologies.  Consideration for the number of inhabitants potentially affected and type of economic activity in the area affected by flood risks as part of the potential adverse consequences associated with flood scenarios.  Importance of consistency between the information in maps of flood hazards and risks relevant information in the WFD.  Adaptation of management plans depending on scientific and technical progress.  Need for member States to make available to the public the preliminary flood risk assessment, maps, and management plans.   \| Respect of the Charter of Fundamental Rights of the European Union which includes, among others: everyone has the right to the protection of personal data concerning him or her (Article 8), the freedom of expression and information (Article 11) \| \| --- \| | **Risk and materialized impacts:**  Recognition that floods can compromise economic development and undermine economic activities due to their potential to cause fatalities, displacement of people, and damage to the environment.  **Risk management:**  Measures should prevent and reduce damage to human health, the environment, cultural heritage, and economic activity, and if possible reduce the likelihood of flooding with, for example, the promotion of sustainable land use practices, the improvement of water retention, and controlled flooding in certain areas.  Need for “fair sharing of responsibilities” (p. 28) for measures jointly decided for the common benefit of the Community in light of the solidarity principle.   \| Choice based on the WFD in case of multi-purpose use of bodies of water for different forms of sustainable human activities (the most stringent objective shall apply unless it is disproportionately expensive or infeasible under some conditions); some possible exemptions from the objectives of ‘good status’ or ‘non-deterioration’. \| \| --- \|  \| Respect of the Charter of Fundamental Rights of the European Union which includes, among others, the right to life for everyone (article 2), the right to property (article 17), the prohibition of discrimination based on grounds such as sex, race, color, ethnic or social origin, genetic features, language, religion or belief, political or any other opinion, membership of a national minority, property, birth, disability, age or sexual orientation (article 21), the equality between women and men in all areas (article 23), and the right to fair just and healthy working conditions (article 31). \| \| --- \| | **Harmer/Harmed:**  Some human activities and climate change contribute to an increase in the likelihood and adverse impacts of flood events.  **Corrective actions:**  Possibility to grant rapid financial assistance through the European Union Solidarity Fund to help people to return to conditions as normal as possible.   \| Based on the WFD: recovery of costs based on the polluter-pays principle, and considering its social, environmental, and economic effects.  Restoration of all bodies of surface- and ground-waters to good water status.  Note: in the WFD, floods are considered as a threat to spread pollution and affect negatively the water status. \| \| --- \| |

|  | **Procedural** | **Distributive** | **Corrective** |
| --- | --- | --- | --- |
| **Ecological** | **Participation:**  NOT INCLUDED  **Information:**  Consideration of the potential sources of environmental pollution as a consequence of floods in different flood scenarios.  Assessment of effects of the measures on the environment.   \| According to the WFD, the vulnerability of aquatic ecosystems should be taken into account for effective and coherent water policy. \| \| --- \| | **Risk and materialized impacts:**  Recognition that floods damage the environment. If they threaten an unpopulated area with limited ecological value, then the risk is considered not to be significant.  **Risk management:**  Importance that measures reduce and prevent damage to, among others, the environment and promote the achievement of environmental objectives laid down in the Community legislation.  Consideration for giving more space to rivers and using some floodplains as natural retention areas.  Development of river basin management plans for each river basin district in order to achieve good ecological and chemical status which contribute to mitigate the effects of floods.  Integrated river basin management with the WFD.   \| Based on the WFD for environmental objectives. Possibility of exceptional exemptions based on specific conditions.  Looking for best environmental practices.  Integrating qualitative and quantitative aspects for the purpose of environmental protection.  Protecting aquatic and terrestrial ecosystems and wetlands. Including wise use and conservation of wetlands because of their functions to protect water resources. Including conservation of habitats and species directly depending on water as well. \| \| --- \|  \| Respect of the Charter of Fundamental Rights of the European Union which includes, among others, environmental protection in accordance with the principle of sustainable development (article 37). \| \| --- \| | **Harmer/Harmed:**  NOT INCLUDED  **Corrective actions:**  Possibility to grant rapid financial assistance through the European Union Solidarity Fund to help natural zones to return to conditions as normal as possible.  Where possible, maintenance and restoration of floodplains.   \| Based on the WFD: recovery of costs, including environmental and resource costs associated with damage or negative impact on the aquatic environment, based on the polluter-pays principle, and considering, among others, the environmental effects of recovery. \| \| --- \| |

|  | **Procedural** | **Distributive** | **Corrective** |
| --- | --- | --- | --- |
| **Spatial** | **Participation:**  Responsibility of Member States for flood risk management on their own territories.  Relevant coordination within river basin districts, with as much as possible a single plan per basin district (with additional plans at the sub-level if appropriate).  Importance of coordinated action at the European Union Community level to improve flood protection. Need for coordination with third countries as well.  Application of the subsidiarity principle as well as the proportionality principle.  In case a Member State identifies an issue that it cannot resolve by itself, then it makes a report to the Commission and other concerned Member States can advise.  **Information:**  Objectives based on local and regional circumstances, plans taking into consideration particular needs and priorities of the specific areas.  Exchange of information between the Member States, especially for international river basins.  Consideration of the appropriate scale for maps, for instance flood hazard maps and flood risk maps covering the geographical area that is potentially affected.  Importance of assessing transnational effects in the analysis of costs and benefits.  Consideration of issues such as topography, position of water courses, and their hydrological and geomorphological characteristics in the assessment of the potential adverse consequences of future floods. | **Risk and materialized impacts:**  Recognition that different types of floods affect different places in the Community. The damage caused by flood events and the causes and consequences of floods vary across countries and regions.  **Risk management:**  Coordination at the river basin level to implement effective measures, taking into account the particular characteristics of the basin or sub-basin.  Importance of the solidarity principle in the context of flood risk management:   - In particular, a Member State should not take measures to reduce the flood risk on its territory if these actions at the same time increase flood risk on another Member State’s territories, unless there is a coordinated and agreed solution between the concerned States. - Encouragement of fair sharing of responsibilities in case measures are jointly decided for the common benefit (such as flood risk management along water courses). - Possible support and assistance from other Member States in major emergencies. | **Harmer/Harmed:**  NOT INCLUDED  **Corrective actions:**   \| Based on the WFD: consideration of geographic and climatic conditions of the region or regions affected with recovery. \| \| --- \|   Possibility to grant rapid financial assistance through the European Union Solidarity Fund to help regions and countries to return to conditions as normal as possible.   \| Respect of the Charter of Fundamental Rights of the European Union: freedom of movement and residence. Every citizen of the Union has the right to move and reside freely within the territory of the Member States (article 45). \| \| --- \| |

|  | **Procedural** | **Distributive** | **Corrective** |
| --- | --- | --- | --- |
| **Temporal** | **Participation:**   \| Respect of the Charter of Fundamental Rights of the European Union: Children shall have the right to such protection and care as is necessary for their well-being. They may express their views freely. Such views shall be taken into consideration on matters which concern them in accordance with their age and maturity (article 24). \| \| --- \|   **Information:**  Consideration of the long-term developments, in particular the likely impact of climate change on the occurrence of floods, including in the reviews of flood hazard and flood risks maps.  Consideration for adverse consequences of future floods for human health, the environment, cultural heritage, and economic activity.  Consideration of past floods and their impacts on human health, the environment, cultural heritage, and economic activity, as well as the likelihood of similar events in the future. | **Risk and materialized impacts:**  Increasing likelihood and adverse impacts of flood events due to climate change.  **Risk management:**  Focus on prevention, protection, and preparedness, including flood forecasts and early warning systems.  Regular reviews and updates of the flood risk management plans.   \| Respect of the Charter of Fundamental Rights of the European Union which includes, among others, responsibilities and duties with regard to future generations (preamble). \| \| --- \| | **Harmer/Harmed:**  NOT INCLUDED  **Corrective actions:**   \| Based on the WFD:  The choice of recovery actions needs an economic analysis of water services based on long-term forecasts.  Measures to face exceptional circumstances should not compromise the recovery of the quality of the bodies of water once the circumstances are over. \| \| --- \| |
